# Inhibitory effects of epigenetic modulators and differentiation inducers on human medulloblastoma cell lines

**DOI:** 10.1186/1756-9966-32-27

**Published:** 2013-05-14

**Authors:** Ina Patties, Rolf-Dieter Kortmann, Annegret Glasow

**Affiliations:** 1 Department of Radiation Therapy, University of Leipzig, Stephanstraße 9a, Leipzig, 04103, Germany

**Keywords:** Medulloblastoma, 5-Aza-2’-deoxycytidine, Resveratrol, Abacavir, Valproic acid, Retinoic acid, SAHA

## Abstract

**Background:**

Medulloblastoma (MB) is the most common malignant brain tumor in childhood with a 5-year survival of approximately 60%. We have recently shown that treatment of human MB cells with 5-aza-2’-deoxycytidine (5-aza-dC) reduces the clonogenic survival significantly. Here, we tested combinatorial effects of 5-aza-dC with other epigenetic (valproic acid, SAHA) and differentiation-inducing drugs (resveratrol, abacavir, retinoic acid) on human MB cells *in vitro* to intensify the antitumor therapy further.

**Methods:**

Three human MB cell lines were treated with 5-aza-dC alone or in combination for three or six days. Metabolic activity was measured by WST-1 assay. To determine long-term reproductive survival, clonogenic assays were performed. Induction of DNA double-strand break (DSB) repair was measured by γH2AX assay.

**Results:**

The applied single drugs, except for ATRA, reduced the metabolic activity dose-dependently in all MB cell lines. Longer treatment times enhanced the reduction of metabolic activity by 5-aza-dC. Combinatorial treatments showed differential, cell line-dependent responses indicating an important impact of the genetic background. 5-Aza-dC together with resveratrol was found to exert the most significant inhibitory effects on metabolic activity in all cell lines. 5-aza-dC alone reduced the clonogenicity of MB cells significantly and induced DSB with no further changes after adjuvant administration of resveratrol.

**Conclusion:**

The observed significant decrease in metabolic activity by combinatorial treatment of MB cells with 5-aza-dC and resveratrol does not translate into long-term reproductive survival deficiency *in vitro*. Further studies in animal models are needed to clarify the resveratrol-mediated anticancer mechanisms *in vivo*.

## Background

Medulloblastoma (MB) is the most common malignant brain tumor in childhood and accounts for 20% of such entities. It arises during embryonic development from neural precursor cells in the precerebellum or the dorsal brain stem [1]. It is widely believed that genetic, gene regulatory, or epigenetic abnormalities give rise to tumor initiation and inhibit normal neuronal or glial differentiation in neural stem cells [2]. MB standard therapy includes primary tumor resection followed by irradiation and/or chemotherapy. At the moment, therapy stratification depends on tumor histology, metastasis stage, and patient age. Patients belonging to the high-risk group and such with metastases receive a more intensive concomitant chemoradiotherapy compared to low-risk patients. Infants below 18 months do not obtain radiation therapy to avoid radiation-related adverse late effects, like neurocognitive and psychomotoric deficits, but receive a highly aggressive chemotherapy. With overall 5-year survival rates of approximately 60%, an improved antitumor strategy is urgently needed to further enhance the outcome of the moderate- and high-risk patients (90% of all MB patients). Especially in younger children, a reduction of treatment-induced adverse effects, by applying less toxic agents, is an ambitious aim in MB therapy optimization.

Epigenetic aberrations like *HIC1*, *RASSF1a*, or *CASP8* promoter methylation, which are observed in most MBs (70–90%), lead to silenced tumor suppressor genes (TSG) and are responsible for the lack of cell cycle arrest and apoptosis in tumor cells [2]. Hence, the application of epigenetic modulators in the treatment of MB might be a suitable approach to improve the standard therapy. Methyltransferase inhibitors like 5-aza-2’-deoxycytidine (5-aza-dC, decitabine) and histone deacetylase inhibitors (HDACi) like valproic acid (VPA) or SAHA are approved for the therapy of other diseases such as myelodysplastic syndromes, neurological disorders, or T-cell lymphoma. Application of epigenetic drugs in leukemia and carcinomas is currently tested in clinical studies. In addition, the low differentiation stage of MB cells constitutes also an attractive approach for MB therapy. The usage of differentiation-inducing drugs may induce neuronal or glial maturation in tumor cells and, therefore, eliminate their cancer-causing abilities. For instance, all-*trans* retinoic acid (ATRA) has already been used in differentiation therapy of leukemia patients. *In vitro* experiments with abacavir and resveratrol exhibited the drug-mediated induction of a more differentiated cell phenotype in MB cell lines [3-5]. Combination of nucleoside analogs like 5-aza-dC with HDACi might result in amplified effects as HDACi have been shown to suppress the alien nucleotide removal [6]. Also, induction of differentiation might work much more successfully after reactivation of beforehand silenced differentiation-relevant genes [7].

In this study, we tested single and combinatorial effects of 5-aza-dC with other epigenetic drugs (VPA, SAHA) or differentiation inducers (resveratrol, abacavir, ATRA), as detailed below, on the metabolic activity and reproductive survival of human MB cell lines.

5-Aza-dC, a cytidine analog and methyltransferase inhibitor, is approved by the U.S. Food and Drug Administration (FDA) for the treatment of myelodysplastic syndrome since 2006. 5-Aza-dC is known to reactivate silenced TSG by demethylation of their promoter regions in MB and other tumor cells after incorporation into the DNA during the replication process [8-10]. DNA-integrated 5-aza-dC traps *de novo* methyltransferases (DNMT) and induces DNA damage including double-strand breaks (DSB) [11,12]. We have recently shown that 5-aza-dC treatment of human MB cells reduces their vitality, proliferation rate, and clonogenic survival significantly [8]. Others have described similar effects in leukemia and lung cancer cell lines [13,14].

VPA, an HDACi, has already been established in the treatment of epilepsy and depression, and clinical trials for its application in HIV and cancer patients are ongoing. VPA leads to hyperacetylation of histone proteins resulting in activation of cell cycle arrest and apoptosis in human MB cells [15]. In xenograft MB mouse models, it was shown that VPA alone reduces tumor growth and prolonges survival [16]. It was also reported that combinatorial treatment with 5-aza-dC and VPA is able to diminish tumor initiation in a *Ptch*-deficient MB mouse model [17].

SAHA (vorinostat, Zolinza™) is the first HDACi approved by the FDA for cancer treatment. SAHA directly interacts with the catalytic domain of histone deacetylases [18]. As a result, gene promoter-bound histones stay hyperacetylated and facilitate the selective transcription of genes [19]. Additionally, SAHA exerts chemosensitizing effects in oral squamous cell carcinoma and medulloblastoma cells [20,21].

Abacavir, a 2-deoxyguanine analog, is approved for HIV and AIDS therapy in the EU since 1999. Two ways of an abacavir-mediated reduction of telomerase activity are reported: 1) indirect, by incorporation into the DNA strand which leads to polymerization stop [22], and 2) direct, by downregulation of *hTERT* (human gene for telomerase reverse transcriptase) mRNA transcription [3]. In recent years, abacavir attracted attention for cancer therapy for its ability to inhibit telomerase activity, which is known to be overexpressed in the vast majority of cancers [23]. Also in 70% of MBs, telomerase activity is enhanced in contrast to normal cerebellum [24]. It was previously shown that treatment of human MB cell lines with abacavir results in proliferation inhibition and neuronal differentiation [3].

ATRA is the prototype of differentiation therapy in cancer cells and, therefore, it is approved for treatment of acute promyelocytic leukemia (APL) in the EU since 1996. Inhibition of proliferation and induction of apoptosis and differentiation have been observed in many tumor cells including MB cells after treatment with ATRA [25-30].

Resveratrol, a plant polyphenol, is described to exhibit tumor-preventive as well as anticancer effects dependent on concentration, cell type, and microenvironment [31-33]. In MB cells, resveratrol has been shown to induce cell cycle arrest, apoptosis, and/or neuronal differentiation [4,5,34].

In this study, we show that the applied single mediators, except for ATRA, reduce the metabolic activity in all MB cell lines. In combinatorial treatments with the epigenetic modifier 5-aza-dC, resveratrol reveals the strongest decrease in metabolic activity, but it can not further reduce the 5-aza-dC-induced decrease of clonogenic survival.

## Methods

### Modulators

5-Aza-2’deoxycytidine (decitabine, trade name Dacogen®), all-*trans* retinoic acid (ATRA), resveratrol, and valproic acid were purchased from Sigma-Aldrich (Munich, Germany). Abacavir hemisulfate was kindly provided from GlaxoSmithKline (Hamburg, Germany) and suberoylanilide hydroxamic acid (SAHA, vorinostat, trade name Zolinza®) from MSD (Haar, Germany). Stock solutions were prepared as follows and stored at - 20°C: 10 mM 5-aza-dC in PBS; 500 μM ATRA in 10% ethanol (stored at - 80°C); 500 μM resveratrol in 1% ethanol; 1 M valproic acid in PBS; 100 mM abacavir in PBS; 100 μM SAHA in 0.25% DMSO. Further work solutions were made in PBS and administered in equal dilutions to the cell medium. To exclude effects based on ethanol or DMSO applications, appropriate controls were implemented.

### Cell lines and cell culture

The human MB cell line MEB-Med8a was kindly provided by Prof. T. Pietsch (Department of Neuropathology, University of Bonn Medical Centre, Bonn, Germany). The MB cell lines D283-Med and DAOY were purchased from ATCC cell biology collection (Manassas VA, USA). D283-Med and DAOY were maintained in MEM (Sigma-Aldrich, Munich, Germany) including 2 mM L-glutamine (Biochrom, Berlin, Germany), MEB-Med8a in DMEM with 4.5 g glucose (Lonza, Basel, Switzerland), all supplemented with 10% FCS (PAA, Yeovil, Somerset, UK), 100 U/ml penicillin, and 100 μg/ml streptomycin (Biochrom, Berlin, Germany) at 37°C and 5% CO_2_ unless otherwise noted.

### Metabolic activity

To examine metabolic activity, cells were seeded in triplicates in 96-well plates, and after 24 h cells were grown with or without the modulator for three or, in case of 5-aza-dC, for three and six days. Combinatorial treatments were executed with/without 3 μM (D283-Med) or 5 μM (DAOY, MEB-Med8a) 5-aza-dC and the second drug (concentrations listed in Table 1). After incubation, medium was discarded, and cells were incubated with normal medium including 10% WST-1 reagent (Roche, Basel, Switzerland) for 1–2 h. Metabolically active cells have the ability to metabolize the tetrazolium salt WST-1 into a formazan dye. The amount of formed formazan dye directly correlates with the number of viable cells. Measuring the formazan dye extinction at 450 nm wave length relative to medium control corresponds to the metabolic activity of the viable cells. IC 30 values were calculated by generating an exponential or linear trend using Microsoft Excel 2003 software.

**Table 1:** Concentrations for the combinatorial treatment with 5-aza-dC

**Modulator**	**D283-Med**	**DAOY**	**MEB-Med8a**
**5-Aza-dC**	3 μM	5 μM	5 μM
**VPA**	0.77 mM	0.77 mM	0.77 mM
**SAHA**	0.16 μM	0.16 μM	0.16 μM
**Abacavir**	0.11 mM	0.11 mM	0.11 mM
**Retinoic acid**	0.25 μM	0.25 μM	0.25 μM
**Resveratrol**	15 μM	15 μM	40 μM

### Clonogenic survival

For clonogenic assays, cells were treated with/without 3 μM (D283-Med) or 5 μM (DAOY, MEB-Med8a) 5-aza-dC in cell culture flasks for three days. Subsequently, medium was renewed and supplemented with 5-aza-dC and 15 μM (D283-Med, DAOY) or 40 μM Resveratrol (MEB-Med8). After three days, cells were counted, seeded at three different cell densities in duplicates in 6-well cell culture plates, and normal medium without mediators was added. Ten to 14 days later, colonies were washed with PBS, fixed with ice-cold ethanol/acetone (1 : 1) for 10 min, stained with Giemsa solution (1 : 1 with distilled water) for 5 min, and washed with distilled water. Colonies with > 50 cells were counted indicating plating efficiency (PE). The ratio between PE of treated cells and PE of untreated cells represented the surviving fraction (SF) of clonogenic cells.

### Statistics

Statistic analyses of were performed using the parametric, one-way, and paired Student’s *t*-test with Microsoft Excel 2003 software. P-values ≤ 0.05 (*) were considered as statistically significant and p-values ≤ 0.001 (**) as highly statistically significant.

Detailed drug interaction analyses regarding synergistic or additive effects were conducted using the Bliss independence (BI) model which is based on the non-interaction theory. The BI model compares the estimates of the combined effects calculated on the individual drug effects with those obtained from the experiment. Therefore, the following equation was used: *E*_*i*_ = *E*_*A*_ × *E*_*B*_, where *E*_*i*_ is the estimated amount of metabolic activity of the theoretical combination of substance A and B, and *E*_A_ and *E*_B_ are the experimental rates of metabolic activity of each drug alone. The interaction of both is described by the difference Δ*E* between the estimated and the observed rates of metabolic activity *ΔE* = *E*_*estimated*_ − *E*_*observed*_[35]. The non-parametric approach described by Prichard *et al.* was modified and used to calculate statistical significance of synergism. In each of the three independent experiments, the observed rates of metabolic activity were subtracted from the predicted values, and the average difference of each experiment was calculated. Statistically significant synergy was claimed when the average difference as well as its 95% confidence interval was positive [36].

## Results and discussion

To determine submaximal concentrations for the inhibition of the metabolic activity of MB cells, we performed incubation experiments with the single drugs. The mean drug concentration of the three examined cell lines which inhibits the metabolic activity by 30% (IC30) was chosen for combination treatments with 5-aza-dC for three days (Table 1). The long-time reproductive cell survival of the combination showing the most promising results in metabolic inhibition was investigated and induction of DSB as a potential mechanism for cell death was evaluated.

### Effects on metabolic activity (WST-1 assay)

**Figure 1:**
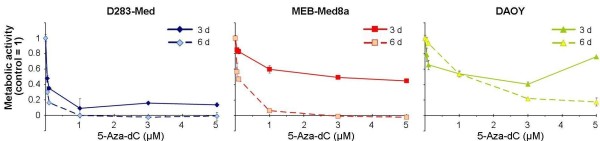
**Time- and dose-dependent inhibition of metabolic activity by 5-aza-dC.** Metabolic activity of three medulloblastoma cell lines was measured by WST-1 assay after 5-aza-dC treatment for three or six days. Raw values were normalized to untreated control. Data from one experiment are shown as means ± SEM of triplicate samples.

After treatment with 5-aza-dC, we observed an enhanced reduction of metabolic activity in all cell lines treated for six days *versus* three days (Figure 1). As 5-aza-dC incorporation depends on cell cycle progression and proliferation frequency [10], the longer incubation period allows more 5-aza-dC to be incorporated into DNA. Surprisingly, 5-aza-dC exhibited the strongest inhibitory effect in slowly proliferating D283-Med cells, whereas DAOY cells, showing the shortest replication time, were much more resistant. Although 5-aza-dC-induced inhibition was stronger after 6 *versus* 3 days of treatment, leading to a total loss of metabolic activity in D283-Med and MEB-Med8a, about 20% of metabolic activity remained in DAOY cells. The relative 5-aza-dC resistance of DAOY cells *versus* MEB-Med8a and D283-Med cells in mortality and cell growth arrest has already been shown by our workgroup [8]. This indicates that, beside the incubation period-dependent incorporation rate, other mechanisms, like repair efficiency or DNMT activity, are involved in 5-aza-dC-induced cytotoxicity.

**Figure 2:**
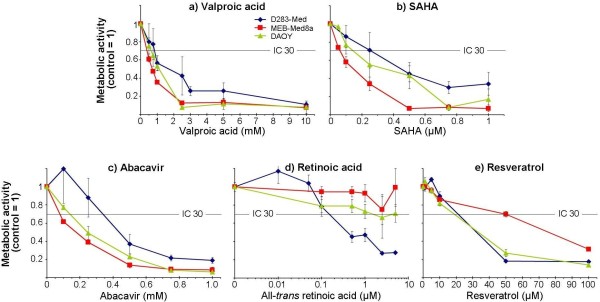
**Dose-dependent inhibition of metabolic activity by valproic acid, SAHA, abacavir, retinoic acid, and resveratrol.** Metabolic activity of three medulloblastoma cell lines was measured by WST-1 assay after treatment with the indicated modulators for three days. Raw values were normalized to untreated control. Data are presented as mean ± SEM from at least three independent experiments done in triplicates. IC 30 values, depicted as black lines, indicate concentrations with 30-percent inhibition of metabolic activity.

**Figure 3:**
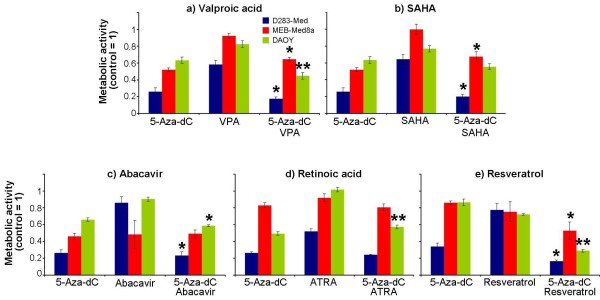
**Combinatorial effects of 5-aza-dC with valproic acid, SAHA, abacavir, retinoic acid, and resveratrol on metabolic activity.** Three medulloblastoma cell lines were treated with 5-aza-dC and/or indicated drugs for three days at concentrations listed in Table 1 and WST-1 test perfomed. Treated samples were normalized to the untreated control. Data show means ± SEM of at least three experiments done in triplicates. The statistical significance of differences between 5-aza-dC and combinatorial treatments is indicated by asterisks: *, p ≤ 0.05; **, p ≤ 0.001.

VPA led to a strong dose-dependent decrease of metabolic activity in all three MB cell lines (Figure 2a). The individual VPA concentrations leading to 30% inhibition (IC 30) were between 0.27 mM (MEB-Med8a) and 0.9 mM (D283-Med) after VPA treatment for three days. After combinatorial treatment with 5-aza-dC, additive effects on the reduction of metabolic activity in two cell lines (DAOY, D283-Med) with a significant synergistic response in DAOY cells were observed. This is in accordance with data obtained from Yang *et al.* showing synergistic effects on inhibition of cell growth and induction of apoptosis in human leukemic cell lines [37]. In contrast, combined 5-aza-dC/VPA treatment of MEB-Med8a cells revealed a significant increase of 25% in metabolic activity compared to 5-aza-dC monotherapy (Figure 3a). Conceivably in MEB-Med8a cells, VPA mainly induces G1 arrest by induction of *p21* expression [15] and, therefore, prevents cytotoxic 5-aza-dC incorporation into the DNA molecule.

Also, SAHA induced a concentration-dependent decrease of metabolic activity (Figure 2b). The IC 30 values were 60 nM ‒ 260 nM (MEB-Med8a, D283-Med). After simultaneous treatment with 5-aza-dC, the metabolic activity of D283-Med and DAOY cells was only slightly reduced, compared to 5-aza-dC alone. Similarly to 5-aza-dC/VPA treatment response, MEB-Meb8a cells exhibited a significant enhancement of metabolic activity after combined treatment with SAHA (Figure 3b). Corresponding to these cell line-specific findings, differential results have also been published showing minor effects in colon carcinoma cells, but significantly enhanced cell death in ovarian cancer and leukemia cells after combinatorial 5-aza-dC/SAHA treatment [38-40].

Treatment of MB cells with abacavir resulted in a dose-dependent reduction of metabolic activity (Figure 2c). Thereby, D283-Med revealed to be the most resistant among the examined cell lines showing an IC 30 value of 340 μM, whereas MEB-Med8a and DAOY cells exhibited IC 30 values of 70 μM and 150 μM. The higher resistance is possibly due to a higher expression of human telomerase reverse transcriptase (hTERT) in D283-Med cells compared to DAOY cells [3,24]. Applying higher abacavir concentrations (350 μM to 750 μM, treated for 24 to 96 h), Rossi *et al.* reported that abacavir induces enhanced mortality in D283-Med cells, but differentiation and growth arrest in DAOY cells [3]. We found here that simultaneous treatment with 5-aza-dC led to an additive response of two MB cell lines (DAOY, D283-Med) in metabolic activity (Figure 3c). This is the first time showing intensifying *in vitro* effects of an epigenetic modifier and a telomerase inhibitor on metabolic activity of tumor cells.

Retinoic acid treatment induced differential, cell line-specific effects: MEB-Med8a cells showed no response to ATRA; DAOY cells exhibited only a moderate reduction of metabolic activity with a maximum of 30%; and in D283-Med cells, a dose-dependent reduction of metabolic activity with up to 70% inhibition could be observed (Figure 2d). This goes along with findings of other groups [28,30,41]. In the highly sensitive D283-Med cell line, an ATRA-mediated caspase 3 induction followed by apoptosis has been reported [28]. In contrast, DAOY cells showed only a moderate ATRA-induced enhancement of caspase 3 expression and apoptosis [30]. The presence of OTX2 (orthodenticle homeobox 2), a homeobox protein acting as a transcription factor during brain development, seems to be necessary for ATRA-induced mortality of tumor cells. In accordance, enhanced OTX2 protein levels have been observed in the sensitive D283-Med cells, whereas the relatively resistant DAOY cells do not express OTX2 [41]. The combinatorial treatment with 5-aza-dC revealed no further effect in the ATRA-sensitive D283-Med cells but led to a significant increase of metabolic activity in DAOY cells compared to 5-aza-dC alone. The simultaneous treatment of the ATRA-resistant MEB-Med8a cells showed no 5-aza-dC-dependent effect on the ATRA responder status (Figure 3d). In contrast, Fu *et al.* reported a 5-aza-dC-induced hypomethylation of the hypermethylated *CRABP-II* (cellular retinoic acid-binding protein) gene promoter in ATRA-resistant MB cells leading to the expression of the afore-silenced gene. This affects the ATRA transport into the nucleus and lead to an ATRA-mediated cellular response in these MB cells [47]. However, the lack of an ATRA response in MEB-Med8a after combined treatment with 5-aza-dC indicates that hypermethylation of the *CRABP-II* promoter is not responsible for ATRA resistance in this MB cell line.

As shown in Figure 2e, resveratrol (> 10 μM) led to a significant concentration-dependent reduction of metabolic activity in all three examined cell lines, possibly by inhibition of STAT3 (signal transducer and activator of transcription 3) expression and activity, which results in irreversible cell cycle arrest or apoptosis [44]. The IC 30 values of 15 μM (D283-Med, DAOY) and 40 μM (MEB-Med8a) are within the concentrations of 40 μM, maximal achievable in blood serum after intravenous injection [42]. The combined administration of resveratrol and 5-aza-dC showed a significant synergistic inhibition of 18% (MEB-Med8a), 41% (D283-Med) and 54% (DAOY) on metabolic activity *versus* 5-aza-dC alone (Figure 3e). The sensitive response of the *TP53*-mutated DAOY cell line might indicate a speculative role of resveratrol in the therapy of highly aggressive and therapy-resistant *TP53*-mutated MB tumors. Numerous studies, regarding the outcome of *TP53*-mutated MBs, which represents about 10% of all MBs, showed a 5-year event-free survival of 0% [43-47]. Interestingly, resveratrol has been shown to induce apoptosis p53-dependently and also p53-independently [48,49].

### Combinatorial effects of 5-aza-dC and resveratrol on clonogenicity and DSB repair

**Figure 4:**
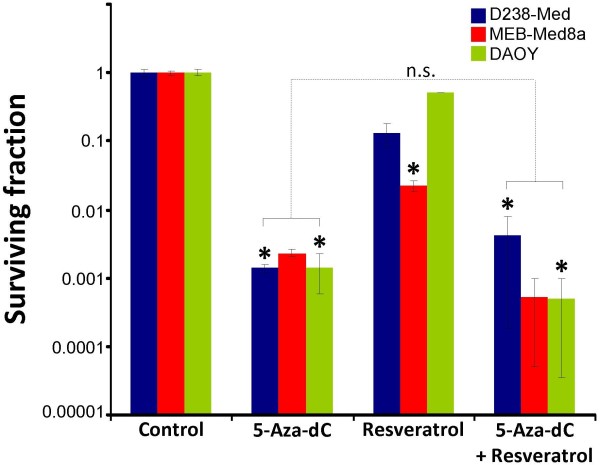
**Clonogenicity after combined treatment with 5-aza-dC and resveratrol.** Clonogenic survival of three medulloblastoma cell lines was determined after treatment with 5-aza-dC and/or resveratrol relative to the untreated control. Surviving fractions from at least two separate experiments done in sextuplicates are depicted and mean values ± SEM are presented. Statistical significance of treated *versus* untreated (control) is indicated by asterisks: *, p ≤ 0.05. Differences between 5-aza-dC and combinatorial treatments are depicted as bracket: n.s. non-significant.

Our investigations on metabolic activity revealed that 5-aza-dC combined with resveratrol achieve the highest antitumor response compared to the other tested drugs. To assess long-time effects, we determined the reproductive cell survival by clonogenic assay after combined 5-aza-dC and resveratrol treatment. 5-Aza-dC alone resulted in a decrease of surviving clonogenic cells exhibiting surviving fractions (SF) between 0.0014 (DAOY, D283-Med8) and 0.0023 (MEB-Med8a), similar to our previously published data [8]. After resveratrol treatment, a significant decline of clonogenic survival was only observed in MEB-Med8a leading to a SF of 0.022, whereas, in DAOY and D283-Med, only small effects were seen (SF_(DAOY)_ = 0.52; SF_(D283-Med)_ = 0.13). The combinatorial treatment with 5-aza-dC and resveratrol revealed no overall decline but cell line-specific effects on clonogenic survival. A resveratrol-mediated enhancement of 5-aza-dC-induced clonogenic cell death was observed in MEB-Med8a and DAOY with a reduction by 78% (SF = 0.0005) and 64% (SF = 0.0005) *versus* 5-aza-dC alone. In contrast, resveratrol showed protective effects on clonogenicity of D283-Med cells represented by a 2.9fold enhancement (SF = 0.0041) in clonogenic survival of 5-aza-dC-treated cells (Figure 4).

**Figure 5:**
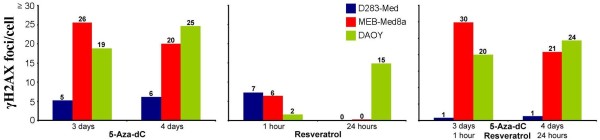
**DSB induction after 5-aza-dC and/or resveratrol treatment.** Induction of DNA double-strand break repair was measured by γH2AX assay in three medulloblastoma cell lines after treatment with 5-aza-dC and/or resveratrol. Numbers of foci per cell less initial number of untreated cells are represented. One experiment was performed containing at least 50 counted cell nuclei.

A common mechanism for the initiation of clonogenic cell death is the induction of DSB [50]. Therefore, we measured the DSB indirectly by immune fluorescence staining of γH2AX repair protein 1 h and 24 h after resveratrol treatment. 5-Aza-dC or resveratrol alone caused the formation of γH2AX foci, although there was no correlation between initial (1 h) nor residual (24 h) foci number and surviving fraction. Palii *et al.* have previously described the DSB-inducing cytotoxic capabilities of 5-aza-dC in cervix and colon carcinoma cells [12]. Also, it was shown that resveratrol influences the DSB repair cascade and, thereby, induces γH2AX foci in ovarian cancer cells [51]. Adjuvant resveratrol administration exhibits no further effects on the 5-aza-dC-induced DSB repair, as no additional foci induction in MEB-Med8a and DAOY cells was found. Contrary to this, in D283-Med cells even a decrease of DSB formation was detected (Figure 5) which is going along with our findings showing an enhancement of clonogenic survival. Moreover, the resveratrol-mediated induction of base excision repair [52] which is shown to be p53-dependent [53], might reduce the priorly DNA-incorporated 5-aza-dC in p53 wild-type D283-Med cells. Possibly, similar mechanisms are responsible for the protective effects of resveratrol on the survival of normal cells after chemotherapeutical treatment [54,55].

## Conclusions

Combination of 5-aza-dC and differentiation-inducing or epigenetic mediators show promising antitumor effects on metabolic activity of MB cells. Cell line-specific results indicate an important impact of the genetic background, which is known to be extremely variable in MBs. Further insight in the acting mechanisms, especially of resveratrol, is needed to evaluate the full potential in antitumor therapy and to translate the synergistic effects on short-term metabolic activity into long-term reproductive survival deficiency.

## Competing interests

The authors declare no conflict of interest.

## Authors’ contribution

IP is participated in the design of the study, carried out the experimental assays and draft the manuscript. AG is participated in conceiving the study and helped to draft the manuscript. RK take part in research instruction and development of the manuscript. All authors read and approved the final manuscript.
